# A Guide for Spatial Omics Technologies: Innovation, Evaluation, and Application

**DOI:** 10.1002/advs.202520806

**Published:** 2026-02-23

**Authors:** Xiaofeng Wu, Weize Xu, Da Lin, Leqiang Sun, Jinxia Dai, Gang Cao

**Affiliations:** ^1^ Faculty of Life and Health Sciences Shenzhen University of Advanced Technology Shenzhen China; ^2^ Department of Genetics Stanford University School of Medicine Stanford California USA; ^3^ Centre for Cell Lineage and Development Guangzhou Institutes of Biomedicine and Health Chinese Academy of Sciences Guangzhou China; ^4^ State Key Laboratory of Agricultural Microbiology Huazhong Agricultural University Wuhan China; ^5^ College of Veterinary Medicine Huazhong Agricultural University Wuhan China; ^6^ Faculty of Life and Health Sciences Shenzhen‐Hong Kong Institute of Brain Science and The Brain Cognition and Brain Disease Institute Shenzhen Institute of Advanced Technology Chinese Academy of Sciences Shenzhen China; ^7^ NMPA Key Laboratory For Research and Evaluation of Viral Vector Technology in Cell and Gene Therapy Medicinal Products Shenzhen Institute of Advanced Technology Chinese Academy of Sciences Shenzhen China

**Keywords:** imaging‐based, multi‐modal, multi‐omics, NGS‐based, spatial omics

## Abstract

Biological macromolecules assemble into sophisticated spatial architectures to orchestrate fundamental cellular processes. Understanding this architecture is therefore essential for deciphering the mechanisms of life. Driven by advances in high‐throughput sequencing and single‐molecule imaging, spatial omics technologies have emerged as powerful tools that are revolutionizing biomedical research. This review systematically evaluates current spatial omics methodologies by comparing their key performance parameters. We critically assess their optimal applications and discuss strategies to overcome prevailing challenges in spatial resolution, capture efficiency, robustness, data analysis, and clinical translation. Furthermore, we highlight how these technologies provide unique insights into tissue heterogeneity, cell‐cell interactions, developmental dynamics, microenvironmental composition, and neuroanatomy. Our analysis offers guidance for selecting appropriate spatial omics approaches and outlines promising directions for future technological innovation and expanded biomedical applications.

## Graphical abstract

This review presents a strategy‐centric framework for spatial omics technologies, organizing methods by how spatial information is experimentally encoded. It compares key performance trade‐offs across sequencing‐ and imaging‐based approaches, examines computational and practical limitations, and highlights biomedical applications. The analysis provides guidance for selecting appropriate technologies and outlines future directions toward higher resolution, scalability, and multimodal integration.

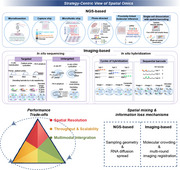

## 1 Introduction

The spatial organization of molecular and cellular components underlies the functional architecture of biological systems [1]. Although conventional omics technologies provide comprehensive molecular inventories, they lack spatial context. This limitation has motivated the rapid development of spatial omics, which maps molecular information directly within intact tissues [2]. These technologies have fundamentally opened up unprecedented vistas into the architecture of life, including tumor microenvironments, intricate neural circuitry, spatiotemporal immune dynamics, and developmental trajectories [3, 4].

**FIGURE 1:**
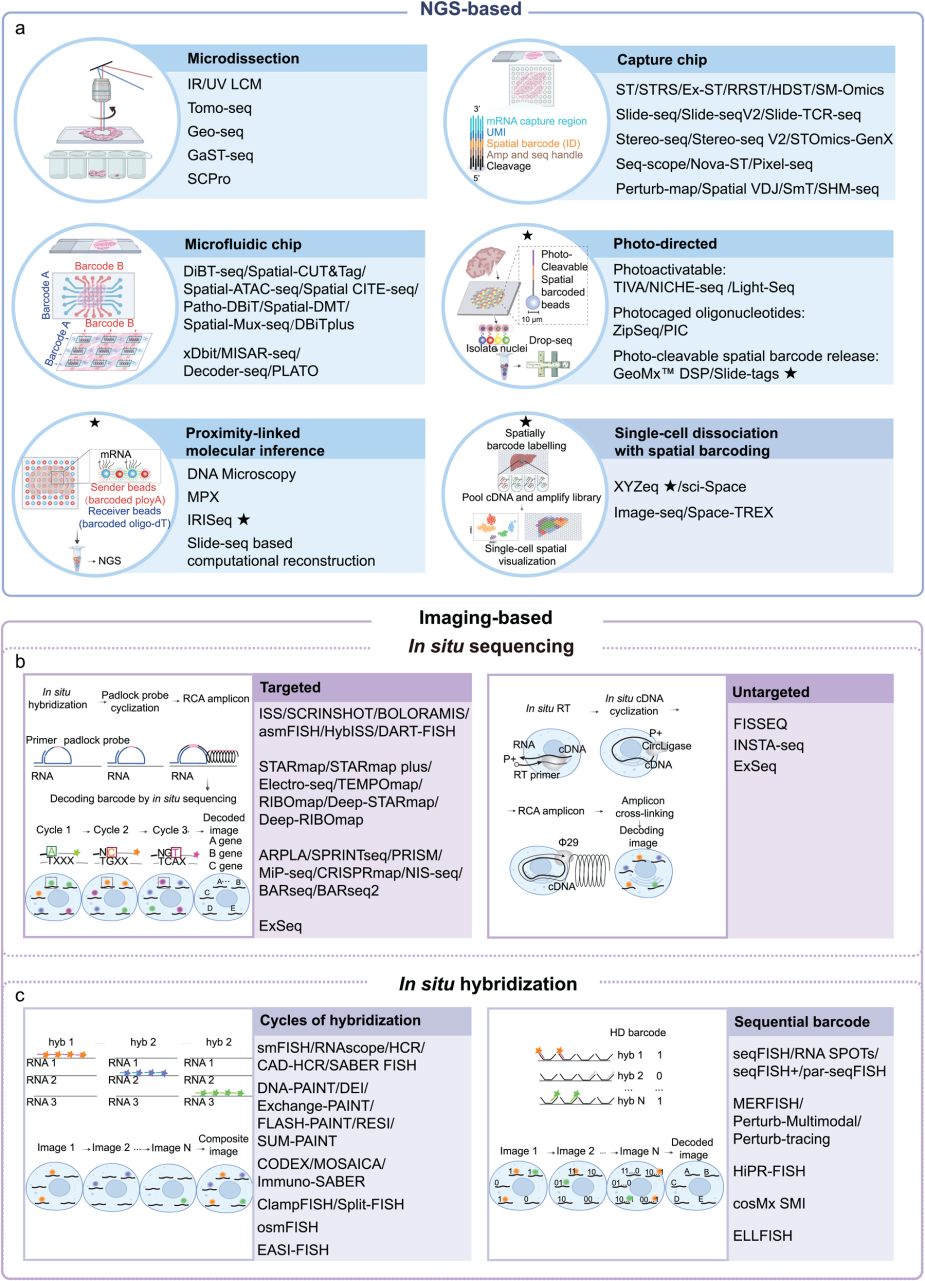
Classification of spatial omics technologies. Spatial omics technologies can be generally classified into NGS‐based methods and imaging‐based methods. (a) NGS‐based methods are further subdivided into distinct subcategories according to their operational principles, including microdissection‐based, capture chip‐based, microfluidic chip‐based, photo‐directed (photoactivatable‐based, photocaged oligonucleotides‐based, photo‐cleavable spatial barcode release), proximity‐linked molecular inference‐based, and single‐cell dissociation with spatial barcoding‐based. Based on spatial barcoding strategies, imaging‐based spatial omics technologies are categorized into: (b) in situ hybridization (ISH)‐based and (c) in situ sequencing (ISS)‐based approaches. Representative methods under each subcategory are listed. The schematic diagram of the principles for each method subclass is shown on the left. For subclass methods whose schematic diagrams cannot be uniformly displayed, only representative methods marked with an asterisk are shown. This classification does not include mass spectrometry‐based proteomics or metabolomics detection methods. Schematic diagrams illustrating photo‐directed and single‐cell dissociation with spatial barcoding‐based approaches were adapted with permission from ref. [6] (Copyright 2023, Springer Nature) and ref. [7] (Copyright 2021, AAAS), respectively. Schematics for proximity‐linked molecular inference and untargeted in situ sequencing‐based approaches were created by the authors, based on the conceptual frameworks of IRISeq [8], FISSEQ [9] and ref. [10].

Spatial omics technologies can be broadly categorized into next‐generation sequencing (NGS)‐based and imaging‐based approaches according to their underlying operational principles (Figure 1) [5]. NGS‐based methods typically rely on spatially barcoded microarrays to enable comprehensive molecular profiling, including whole‐transcriptome analysis. These methods, however, are often limited by coarse spatial resolution and reduced detection efficiency, resulting in many‐to‐one mappings of molecules onto spatial spots, bins, or pixels. In contrast, imaging‐based approaches achieve higher spatial precision and detection fidelity through direct molecular visualization, generally following a one‐to‐one molecular‐to‐pixel mapping paradigm, albeit at the expense of throughput and scalability.

In this review, we adopt a strategy‐centric framework to systematically analyse spatial omics technologies according to their operational principles and coupled performance constraints. Building on this classification, we focus on key performance metrics as composite and context‐dependent properties, and dissect how these properties are shaped by experimental strategies such as spatial barcoding, probe chemistry, tissue processing, molecular diffusion, and computational reconstruction. Rather than treating spatial omics technologies as competing solutions, we highlight how different approaches can be strategically combined to address specific biological questions. Finally, we discuss current technical challenges and emerging directions, outlining how advances in experimental design, instrumentation, and data integration may reshape the landscape of spatial omics (Table S1).

## 2 How Spatial Omics Technologies Work: A Strategy‐Based Classification

### 2.1 Next‐Generation Sequencing (NGS)‐Based Approaches

NGS‐based spatial omics technologies are mainly classified according to how spatial information is experimentally encoded into molecular readouts, including microdissection‐based methods, capture chip‐based approaches, microfluidic chip‐based platforms, photo‐directed techniques, proximity‐linked molecular inference‐based strategies, and single‐cell dissociation coupled with spatial barcoding (Figure 1a). Among these approaches, capture chip‐based methods enable in situ spatial transcriptomic profiling at spot resolution, where a spot denotes the sampling geometry of the minimal capture unit. Detection efficiency is primarily determined by experimental protocols, capture probe density, and sequencing depth. Microfluidic chip‐based platforms utilize orthogonal microfluidic channels to achieve precise pixel resolution barcoding and facilitate integrated multi‐omics analyses through the simultaneous capture of information from different classes of macromolecules. In contrast, photo‐directed methods employ laser‐based activation or cleavage to spatially barcode predefined regions of interest (ROIs) or individual cells, enabling spatial transcriptomic profiling in living or ex vivo tissues. Collectively, these approaches offer complementary advantages in spatial resolution, throughput, and multi‐omics capacities, thereby providing flexible solutions for spatial molecular profiling in diverse biological contexts.

#### 2.1.1 Microdissection‐Based Approaches

Microdissection‐based spatial omics methods integrate microdissection with NGS to enable molecular profiling of spatially defined cell populations (Figure 1a). These methods offer strong sample compatibility, transcriptome‐wide analysis capability, and multi‐omics integration. Representative technologies include laser capture microdissection (LCM) [11, 12], tomo‐seq for low spatial resolution 3D transcriptome mapping [13], geographical position sequencing (Geo‐seq) for single‐cell 3D transcriptomic profiling [14], grid‐assisted spatial transcriptome sequencing (GaST‐seq) for simplified library construction [15], and spatial and cell‐type proteomics (SCPro) for spatial and cell‐type proteomics [16]. However, they still face limitations including relatively low throughput, technical complexity, high costs, target degradation, and high dependency on prior knowledge for regions of interest (ROIs) selection.

#### 2.1.2 Capture Chip‐Based Approaches

Capture chip‐based spatial omics platforms (Figure 1a) primarily employ spatially barcoded microarray chips to capture in situ molecular information from tissue sections. Accordingly, hardware parameters such as microarray spacing, minimum feature size, and capture probe density largely determine key performance metrics including spatial resolution, capture efficiency, and in situ information fidelity. To achieve higher performance, various chip fabrication methods have been developed, such as spot arrays, bead arrays, and high‐density clusters.

Spot array‐based capture technologies (e.g. spatial transcriptomics (ST), expansion spatial transcriptomics (Ex‐ST), high‐definition spatial transcriptomics (HDST), spatial multi‐omics (SM‐Omics)) represent the earliest implementations of capture chip‐based strategy. The original ST platform, later commercialized as Visium, achieves a spatial resolution of approximately 55‐100 µm with a capture efficiency of ∼6.9% relative to single‐molecule FISH (smFISH) [17]. Recent extensions, like Spatial Total RNA Sequencing (STRS), have extended its capabilities to comprehensive RNA species profiling beyond polyadenylated transcripts [18]. Subsequent innovations include Ex‐ST, which leverages tissue expansion and clearing to improve effective spatial resolution [19]; RNA‐rescue spatial transcriptomics (RRST), which enhances mRNA recovery from fresh frozen specimens [20]; HDST, featuring 2 µm honeycomb arrays (1.3% efficiency (vs. smFISH)) [21]; and SM‐Omics, which enables automated co‐mapping of transcriptomic and proteomic information [22].

Bead array‐based platforms (Slide‐seq, Slide‐seqV2, Slide‐TCR‐seq) employ monolayer barcoded beads assembled on glass slides to achieve 10 µm resolution [23], with Slide‐seqV2 increasing RNA capture efficiency to approximately 50% of single‐cell sequencing [24]. Building on Slide‐seq, Slide‐TCR‐seq enables simultaneous transcriptome and TCR (T‐cell receptors) profiling in intact tissues [25]. To further enhance spatial resolution, high‐density clusters‐based technologies such as spatial enhanced resolution omics‐sequencing (Stereo‐seq, utilizing 220 nm DNA nanospheres) [26], Stereo‐seq V2 (extended capability for non‐coding RNA and host‐pathogen transcriptome co‐capture) [27], STOmics‐GenX (integrating Stereo‐seq with CRISPRclean for enhanced efficiency) [28], Seq‐Scope (achieving submicron resolution) [29] have emerged, though at substantial sequencing costs. Recent advancements underscore a focus on balancing technical performance with practical implementation. For example, Nova‐ST is designed for cost‐effective profiling of larger tissue sections [30], while Pixel‐seq utilizes low‐cost polony gels, achieving a substantial (e.g., ≥35‐fold) reduction in expense [31], highlight ongoing efforts to balance technical performance with practical implementation requirements.

Beyond transcriptomic profiling, capture chip‐based approaches have been extended to spatial multimodal analyses. Perturb‐map combines CRISPR‐based genetic perturbation with spatial omics technologies to dissect the context‐dependent functional consequences of genetic alterations within the tumor microenvironment [32]. The spatial metatranscriptomics (SmT) platform employs multimodal arrays to co‐profile microbiomes and host transcriptomes in situ [33], while Spatial VDJ enables spatially resolved profiling of T cell receptors (TCR)/ B cell receptors (BCR) profiling on Visium platform, albeit with limited single‐cell resolution [34]. Collectively, these advancements expand the scope of capture chip‐based spatial omics, enabling integrative investigations of infection dynamics, immune responses, and cellular interactions within their native tissue contexts.

#### 2.1.3 Microfluidic Chip‐Based Approaches

Microfluidic chip‐based spatial transcriptomic methods (Figure 1a) utilize orthogonal microfluidic channel arrays to deliver spatial barcodes directly onto tissue sections, offering enhanced sensitivity compared to capture chip‐based approaches. Representative techniques include deterministic barcoding in tissue for spatial omics sequencing (DBiT‐seq) [35], microfluidic indexing based spatial assay for transposase‐accessible chromatin and RNA‐sequencing (MISAR‐seq) [36], dendrimeric DNA coordinate barcoding design for spatial RNA sequencing (Decoder‐seq) [37], and parallel‐flow projection and transfer learning across omics data (PLATO) [38]. Among them, DBiT‐seq achieves a spatial resolution of 10 µm with a detection efficiency of 15.5% (vs. smFISH), serving as a versatile platform for multi‐omics integration despite potential barcode diffusion [35]. Its framework has been expanded to support spatial epigenome profiling (spatial‐CUT&Tag [39], spatial‐ATAC‐seq [40]), spatial resolved proteome‐transcriptome co‐mapping (spatial CITE‐seq [41]), full‐spectrum RNA capture in FFPE sample (Patho‐DBiT [42]), integrated DNA methylation and transcriptome profiling (spatial‐DMT [43]), and multimodal profiling of chromatin features, transcripts, and proteins (spatial‐Mux‐seq) [44]. More recently, DBiTplus enabled integrated whole‐transcriptome and multiplexed protein profiling from the same sample at single‐cell resolution [45]. Parallel advances in other microfluidics‐enabled spatial barcoding strategies have addressed key challenges. For example, multiplexed deterministic barcoding in tissue (xDBiT) increases experimental throughput and capture efficiency on a single chip [46], MISAR‐seq allows simultaneous profiling of chromatin accessibility and transcriptome [36], Decoder‐seq significantly enhances mRNA capture efficiency (68.9% vs. in situ sequencing) [37], and PLATO applies deep learning‐based reconstruction to enhance the effective spatial resolution of spatial proteomics [38]. Collectively, these microfluidic platforms demonstrate superior mRNA capture efficiency and multi‐omics flexibility compared to capture chip‐based methods.

#### 2.1.4 Photo‐Directed Approaches

Several NGS‐based spatial omics methods leverage photo‐directed approaches to achieve spatial barcoding of predefined ROIs in living cells or ex vivo settings. Representative photoactivatable based methods include transcriptome in vivo analysis (TIVA) [47], NICHE‐seq, which integrates in vivo imaging with transcriptomic profiling [48], and Light‐seq, which uses photo‐crosslinking oligonucleotides for covalent spatial barcoding [49]. Related photo‐caged oligonucleotides‐based methods, such as ZipSeq for high‐throughput sequential labeling [50] and photo‐isolation chemistry (PIC) enabling in situ reverse transcription via photo‐caged oligos [51], further expand ROI‐level multiplexing capacity. Despite pioneering live‐cell spatial transcriptomics, photo‐directed approaches are constrained by technical complexity, limited throughput, and intrinsic trade‐offs between spatial precision and sequencing sensitivity, and typically rely on a priori ROIs selection. To extend spatial resolution beyond ROI‐level barcoding, photocleavable tag‐based strategies have been developed to enable higher‐precision spatial transcriptomic labelling [52]. Notably, Slide‐tags further improves experimental flexibility through photocleavable linkers and, when combined with Drop‐seq, achieves single‐nucleus resolution for cell‐type‐specific spatial profiling [6] (Figure 1a).

#### 2.1.5 Proximity‐Linked Molecular Inference–Based Approaches

To address the high costs of current spatial transcriptomic methods, computational algorithms are utilized to reconstruct proximity‐linked molecular spatial localization without direct imaging. DNA microscopy combines in situ UMI labelling and proximity‐based inference for 2D mapping [53], which has been further extended to 3D transcriptome‐wide imaging in zebrafish embryos [54]. Molecular Pixelation (MPX) employs antibody‐DNA conjugates to reconstruct proximal protein networks [55]. Imaging reconstruction using indexed sequencing (IRISeq) reconstructs transcriptomes by decoding encoded bead proximity interactions [8]. Moreover, Slide‐seq based computational approaches leverage molecular diffusion and dimensionality reduction for spatial reconstruction [56]. These computational assisted methods reduce reliance on specialized imaging instrumentation while maintaining high‐resolution tissue mapping.

#### 2.1.6 Single‐Cell Dissociation With Spatial Barcoding‐Based Approaches

The intrinsic resolution limits of chip‐based spatial omics necessitate signal aggregation across neighbouring capture units, leading to transcriptomic averaging and loss of single‐cell heterogeneity. Integrating spatial transcriptomics with scRNA‐seq improves cell‐type annotation, yet relies on independent experiments and indirect inference of spatial cell states. To overcome these constraints, unified scRNA‐seq‐based spatial platforms have emerged. XYZeq combines combinatorial indexing with spatial tagging to achieve ∼500 µm resolution [7], while sci‐Space employs barcoded agarose microarrays (∼73 µm) to retain spatial context prior to scRNA‐seq [57]. These unified platforms preserve both transcriptional and spatial information, overcoming the limitation of traditional chip‐based methods which lack true single‐cell resolution in sequencing. Furthermore, single‐cell sequencing‐based spatial multimodal omics technologies such as Image‐seq [58], and Space‐TREX [59], integrate spatial transcriptomics with functional analyses (e.g., functional imaging, and lineage tracing) to elucidate cellular dynamics, and clonal evolution with spatial resolution. Collectively, these approaches provide a critical bridge between cell‐type definition, cell‐state characterization, and functional interpretation in spatial contexts.

Taken together, NGS‐based spatial omics technologies now encompass diverse methodologies, including microdissection‐, chip‐, and microfluidic‐based systems, each offering distinct trade‐offs among spatial resolution, capture efficiency, and cost. Recent advances, such as photo‐directed barcoding and single‐cell dissociation coupled with spatial barcoding, have improved single‐cell resolution, while proximity‐linked molecular inference‐based spatial reconstruction provides more accessible alternatives. Nevertheless, challenges related to sensitivity, cost, and scalability remain, underscoring the need for continued methodological optimization to enable broader application.

### 2.2 Imaging‐Based Approaches

Imaging‐based spatial omics techniques achieve high‐throughput interpretation by directly labelling target molecules in situ and employing various in situ encoding and decoding strategies. The encoding and decoding strategies refer to the use of nucleotide sequences or temporally ordered fluorescence patterns to uniquely label individual target molecules, followed by sequential imaging‐based readout to decode and identify these molecular barcodes. Consequently, these approaches offer high spatial resolution, 3D profiling, superior in situ information fidelity, and high detection efficiency. However, performance parameters such as signal interpretation accuracy are also constrained by factors inherent to optical imaging‐based decoding strategies, including optical signal crowding, single‐molecule alignment, and cumulative fluorescence background across multiple imaging cycles.

Imaging‐based spatial omics technologies primarily operate through two paradigms: in situ sequencing (ISS) and in situ hybridization (ISH) (Figures 1b,c). ISS methods are subdivided into: (1) targeted approaches, which utilize gene‐specific probes tagged with spatial barcodes and in situ sequencing to decode spatial positions; (2) untargeted approaches, which utilize unbiased in situ cDNA synthesis and enrichment, following by in situ sequencing to determine the gene identity of each transcript by directly reading its cDNA sequence. ISH strategies include cyclic ISH (based on repeated hybridization, imaging, and signal removal) and sequential ISH (using temporal barcoding to reconstruct spatial gene expression). Recent advances have significantly boosted sensitivity, throughput, and multi‐omics integration.

#### 2.2.1 In Situ Sequencing‐Based Approaches

In situ sequencing (ISS)‐based spatial transcriptomics methods have emerged as a powerful approach for high‐fidelity RNA detection. Mats Nilsson's lab pioneered targeted in situ sequencing using barcoded padlock probes (targeting cDNAs of mRNAs) and rolling circle amplification for expression profiles of specific genes harboring SNPs (single nucleotide polymorphism) in breast cancer [60]. By directly targeting RNA with padlock probes and optimizing the SplintR ligation for circulation, subsequent padlock probe‐based methods such as single‐cell resolution in situ hybridization on tissues (SCRINSHOT) [61], barcoded oligonucleotides ligated on RNA amplified for multiplexed and parallel in situ analyses (BOLORAMIS) [62], and amplification‐based single‐molecule fluorescence in situ hybridization (asmFISH) [63] improved specificity and sensitivity. Meanwhile, hybridization‐based in situ sequencing (HybISS) [64] and decoding amplified targeted transcripts with fluorescence in situ hybridization (DART‐FISH) [65] advanced decoding strategies that do not rely on traditional enzymatic in situ sequencing.

To enable spatial transcriptomics in 3D tissues, spatially‐resolved transcript amplicon readout mapping (STARmap) was developed as a hydrogel‐based platform using targeted in situ sequencing to map 28 genes in thick mouse brain sections [66]. Its extension, STARmap PLUS, allowed integrated transcriptome‐proteome profiling [67]. Further innovations include electro‐seq, which incorporates bioelectronic sensing to link gene expression with function [68], TEMPOmap, specializing in nascent RNA [69], and RIBOmap, which targets translating mRNAs [70]. Recent high‐throughput versions, Deep‐STARmap and Deep‐RIBOmap, profile thousands of genes in 200 µm‐thick tissues with improved spatial resolution [71]. Together, these methods form a comprehensive framework for 3D spatial molecular profiling within intact tissue architectures.

Recent advances in ISS‐based spatial omics have expanded in situ sequencing from transcript localization toward integrated molecular and functional profiling. Methodological innovations span three major dimensions. First, ISS has been extended to resolve RNA molecular states and chemical modifications. The sialic acid aptamer and RNA in situ hybridization‐mediated proximity ligation assay (ARPLA) enables single‐cell visualization of glycosylated RNAs, facilitating investigation of RNA glycosylation in physiological and pathological contexts [72]. Second, improvements in sequencing chemistry and encoding strategies have substantially increased throughput and efficiency. Spatially resolved and signal‐diluted next‐generation targeted sequencing (SPRINTseq) enables rapid multiplexed profiling of 108 genes in mouse brain within 48 h [73], while profiling of RNA in situ through single round of imaging (PRISM) enhances imaging throughput through spectral encoding, albeit with limited fluorescence intensity resolution [74]. Third, ISS platforms increasingly support multi‐omics and functional interrogation. Multi‐omics in situ pairwise sequencing (MiP‐seq) enables subcellular detection of DNA, RNA, proteins, and small molecules at single‐base resolution [75]. Integration with perturbation strategies further enables spatially resolved genotype‐phenotype mapping, exemplified by CRISPRmap and nuclear in situ sequencing (NIS‐Seq) [76, 77]. Collectively, these advances transform ISS into a versatile platform for spatially resolved molecular and functional analysis in native tissues.

Building on these advances, targeted ISS platforms have progressively addressed key technical bottlenecks across tissue‐scale, 3D analysis, sensitivity, throughput, and functional integration. In contrast, Untargeted ISS approaches (such as fluorescence in situ sequencing (FISSEQ), in situ transcriptome accessibility sequencing (INSTA‐seq), and expansion sequencing (ExSeq)) employ cDNA circularization with UMIs for iterative barcode decoding (Figure 1b), but remain constrained by low capture efficiency. FISSEQ achieves unbiased profiling of gene expression and RNA processing, but suffers from low mRNA detection (0.005%) due to rRNA dominance (82.7%) [9]. INSTA‐seq improves mRNA detection efficiency and enables mRNA‐RBP interaction visualization, though its capture efficiency still lags far behind single‐cell sequencing methods [78]. For nanoscale resolution, ExSeq combines expansion microscopy with whole‐transcriptome in situ sequencing, enabling high‐resolution mapping of thousands of genes and splice variants in mouse brain tissue [79]. Collectively, these untargeted strategies emphasize resolution and unbiased discovery at the expense of efficiency.

#### 2.2.2 In Situ Hybridization‐Based Approaches

The single‐molecule FISH (smFISH) overcomes the limited sensitivity of most NGS‐based spatial omics approaches by enabling precise and quantitative mapping of individual RNA molecules at single‐cell resolution, albeit with intrinsically low throughput. To improve scalability, cyclic ISH strategies were developed, increasing throughput through iterative hybridization‐imaging cycles combined with signal amplification (Figure 1c). Branched DNA approaches, exemplified by RNAscope and hybridization chain reaction (HCR) enable signal enhancement, while subsequent refinements such as computer‐aided design of reversible hybridization chain reaction (CAD‐HCR) and signal amplification by exchange reaction (SABER), introduced reversible assembly or primer exchange reactions to further improve efficiency, reduce background, and expand multiplexing capacity [80, 81, 82, 83, 84, 85, 86].

Beyond signal amplification, transient binding of fluorescently labelled DNA probes has empowered super‐resolution imaging of subcellular structures. Points accumulation for imaging in nanoscale topography (DNA‐PAINT), DNA Exchange Imaging (DEI), and Exchange‐PAINT [87, 88, 89], achieve nanometer‐scale resolution through stochastic probe exchange, while advanced variants such as fluorogenic labeling in conjunction with transient adapter‐mediated switching for high‐throughput DNA‐PAINT (FLASH‐PAINT), resolution enhancement by sequential imaging (RESI), and secondary label‐based unlimited multiplexed PAINT (SUM‐PAINT) further extend this framework to highly multiplexed in situ analysis at single‐protein resolution [90, 91, 92]. Collectively, these principles have been generalized beyond transcriptomics to proteomic and multi‐omics profiling (e.g., co‐detection by indexing (CODEX) [93], multi‐omic single‐scan assay with integrated combinatorial analysis (MOSAICA) [94], and Immuno‐SABER [95]) and further enhanced through improvements in specificity (e.g., clampFISH [96] and split‐FISH [97]), throughput (e.g., osmFISH), and three‐dimensional imaging of thick tissues (e.g., expansion‐assisted iterative FISH (EASI‐FISH) [98]), substantially broadening the applicability of imaging‐based spatial omics technologies.

The detection throughput of cyclic ISH methods increases linearly with imaging cycles. In contrast, sequential ISH methods enable exponential throughput scaling via temporal encoding strategy, in which high‐throughput molecular profiling at single‐molecular resolution can be achieved in significantly fewer cycles through multi‐round image alignment and temporal barcode decoding (Figure 1c). Leading implementations include sequential FISH (seqFISH) [99], multiplexed error‐robust fluorescence in situ hybridization (MERFISH) [100], CosMx SMI [101], and enhanced electric fluorescence in situ hybridization (EEL FISH) [102], all of which achieve high‐throughput spatial profiling with minimal imaging cycles.

seqFISH has evolved through a series of iterative innovations that progressively expanded its scale, throughput, and biological scope. The original seqFISH introduced temporal combinatorial barcoding to enable highly multiplexed RNA detection by sequential hybridization and imaging [99, 103]. Subsequent developments, including RNA sequential probing of targets (RNA SPOTs), improved transcriptome coverage and imaging efficiency by integrating poly(A)‐based capture strategies [104]. seqFISH+ further scaled the approach to whole‐transcriptome spatial profiling by mitigating signal crowding through expanded combinatorial coding [105]. Beyond transcript detection, seqFISH was extended to multimodal measurements, enabling simultaneous spatial profiling of DNA, RNA, and proteins to interrogate chromatin‐gene expression relationships [106, 107]. More recently, parallel seqFISH (par‐seqFISH) broadened applicability to complex microbial communities [108], while Based on seqFISH (BaseMEMOIR) enabled simultaneous cell lineage trajectory tracking and spatial transcriptomic dynamics mapping [109]. Collectively, these advances have transformed seqFISH from a multiplexed imaging assay into a versatile framework for spatially resolved, multimodal, and lineage‐informed biology.

Continuous innovations in chemistry, optics, and bioinformatics have systematically advanced MERFISH to address fundamental challenges in multiplexing accuracy, signal density, modality integration, and scalability. The original MERFISH framework introduced error‐robust combinatorial barcoding using Hamming codes, establishing highly multiplexed and reliable RNA detection [100]. Subsequent developments improved signal fidelity and tissue compatibility through chemical stabilization, amplification strategies, and integration with expansion microscopy, enabling accurate decoding of densely packed transcripts in intact tissues [110, 111, 112]. MERFISH was further extended beyond transcriptomics to interrogate 3D genome organization, spatial epigenomic features, and thick tissue sections, broadening its applicability to complex biological systems [113, 114, 115]. More recent integrations with genetic perturbation screening enabled simultaneous mapping of gene regulation, chromatin state, and cellular phenotypes in situ [116, 117]. To address scalability and cost, next‐generation implementations, including MERFISH integrated with in situ RNA amplification (RT&T‐AMP) and RAEFISH, substantially reduce probe requirements while enabling whole‐transcriptome and isoform‐resolved spatial profiling [118, 119]. Collectively, these advances position MERFISH as an extensible platform for high‐resolution, multimodal spatial biology.

In addition, CosMx SMI [101] and EEL FISH [102] exemplify innovative solutions via engineering strategies to enhance encoding capacity, detection throughput and signal‐to‐noise ratio for multiplexed spatial analysis. More broadly, sequential ISH strategies enable exponential multiplexing through temporal encoding, overcoming the inherent linear scalability of cyclic ISH. As exemplified by platforms such as MERFISH, CosMx SMI, and EEL FISH, this paradigm supports high‐resolution and error‐robust spatial mapping and has been extended to 3D transcriptomics, multimodal integration, lineage tracing, and perturbation screening in complex tissues.

## 3 A Strategy‐Centric View of Spatial Omics Performance

### 3.1 Spatial Information Capture: Experimental Strategies and Trade‐Offs

**FIGURE 2:**
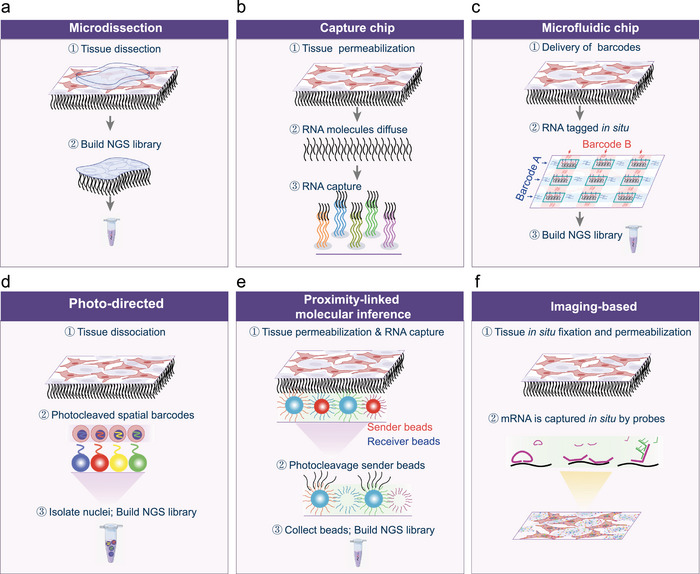
Comparison of spatial capture modalities for spatial omics technologies. Spatial omics technologies employ six distinct molecular capture strategies. (a) Microdissection‐based: Selected ROIs tissues are physically dissected for downstream NGS library construction. (b) Capture chip‐based: Tissue sections are permeabilized, allowing RNA diffusion and subsequent captured by spatially barcoded oligonucleotide probes immobilized on microarray chips. (c) Microfluidic chip‐based: Two‐round orthogonal barcoding delivers unique spatial barcode combinations to each pixel in situ. (d) Photo‐directed‐based: Permeabilized tissues undergo photo‐cleavable barcode release from beads into nuclei, followed by single‐nucleus isolation and NGS library preparation. (e) Proximity‐linked molecular inference‐based: Permeabilized tissues undergo RNA capture using sender/receiver beads. Upon photocleavage, proximity barcodes are released from sender beads and subsequently captured by RNA‐bound receiver beads, enabling reconstruction of spatial relationships prior to NGS. (f) Imaging‐based spatial omics: Fixed/permeabilized tissues are subjected to in situ hybridization or in situ sequencing, followed by high‐resolution microscopy imaging.

Spatial omics methodologies can be organized into six principal molecular capture strategies (Figure 2), which differ fundamentally in how spatial information is physically preserved, molecularly encoded, or computationally inferred. Microdissection‐based approaches obtain spatial context through physical isolation of tissue regions, enabling high‐efficiency molecular recovery comparable to single‐cell or bulk RNA‐seq, but with limited spatial resolution (Figure 2a). Moving from physical separation toward in situ molecular encoding, capture chip‐based strategies rely on solid‐phase‐immobilized probes to anchor transcripts directly within intact tissues, enabling broad‐area coverage. In contrast, microfluidic chip‐based methods extend this paradigm through dual‐round orthogonal barcoding to deliver free probes, significantly improving capture efficiency (Figure 2b,c). Photo‐directed barcode release‐based techniques integrating spatial barcodes with Drop‐seq workflows, can ultimately achieve real single‐cell spatial resolution (Figure 2d).

Beyond direct capture, proximity‐linked molecular inference‐based approaches reconstruct spatial relationships through molecular exchange between sender and receiver beads, inferring spatial proximity without prior imaging and thereby reducing experimental complexity and cost (Figure 2e). Imaging‐based strategies preserve spatial information through direct molecular visualization, leveraging in situ amplification and targeted probe design to maintain native molecular distributions while achieving high spatial precision, single‐cell resolution, and superior detection efficiency (Figure 2f). Collectively, these strategies define a continuum in which spatial precision, molecular coverage, throughput, and operational complexity are traded against one another, providing a unifying framework for selecting spatial omics technologies based on experimental objectives rather than nominal resolution alone.

### 3.2 Factors Determining the Effective Spatial Resolution in Practice

**FIGURE 3:**
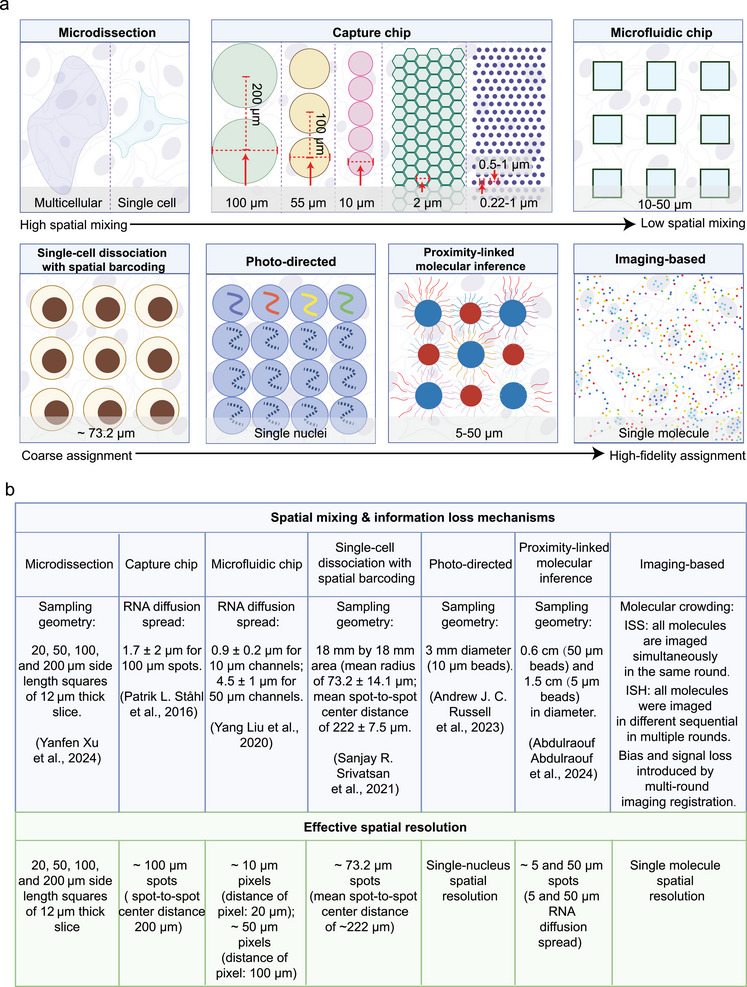
Comparison of spatial resolution across different spatial omics techniques. (a) Spatial resolutions achieved by different spatial omics methodologies. Microdissection‐based methods enable multicellular to single‐cell resolution. Capture chip–based approaches span a wide range of feature sizes, including 100 µm (spot pitch ∼200 µm), 55 µm (spot pitch ∼100 µm), 10 , 2, 0.22 µm (feature spacing ∼0.5 µm), and ∼1 µm (feature spacing ∼0.6–1 µm). Microfluidic chip‐based methods typically achieve effective pixel sizes on the order of ∼10–50 µm (distance of pixel: 20–100 µm). Single‐cell dissociation coupled with spatial barcoding achieves an effective resolution of ∼73.2 µm. Photo‐directed approaches enable single‐nucleus resolution, while proximity‐linked molecular inference‐based methods achieve resolutions of ∼5–50 µm. Imaging‐based methods provide single‐molecule resolution. (b) Spatial mixing levels (this refers to the aggregation of spatially distinct sequence information within a single spot or pixel), mechanisms of spatial information loss, and the resulting effective spatial resolution across different spatial omics technologies, exemplified by representative studies, respectively.

In spatial omics, spatial resolution should not be interpreted solely as the physical dimension of a capture unit or imaging pixel. Rather, it refers to the minimal spatial scale at which molecular signals can be reliably assigned to distinct biological entities, such as individual cells or subcellular compartments. Consequently, spatial resolution represents a composite performance metric jointly shaped by experimental design and computational reconstruction. Its practical upper bound is determined not only by the geometric properties of capture structures, but also by spatial barcoding strategies, tissue permeabilization and molecular diffusion dynamics, as well as downstream cell segmentation and deconvolution algorithms. Importantly, tissue thickness and dimensionality further influence effective resolution: imaging‐based platforms can achieve high‐confidence single‐cell and 3D spatial resolution in thicker specimens by integrating structural markers with advanced computational analysis, whereas NGS‐based methods typically rely on serial tissue sectioning and indirect computational reconstruction to approximate 3D spatial context. These factors collectively give rise to substantial variability in the effective spatial resolution achieved by different spatial omics technologies (Figure 3).

From a technological perspective, microdissection‐based approaches generally achieve multicellular, and in some cases near/single‐cell, spatial resolution (Figure 3). However, the irreversible nature of physical sectioning can disrupt tissue continuity and result in the loss of molecular information at dissection boundaries. Capture chip‐based approaches rely on predefined feature sizes, which impose intrinsic constraints on spatial resolution (Figure 3a). When capture units exceed the dimensions of individual cells, molecular readouts represent averaged signals from multiple neighbouring cells, obscuring cell‐type specific interactions and local microenvironmental features. Conversely, when capture units are smaller than a single cell, computational deconvolution using single‐cell RNA sequencing references becomes necessary to reconstruct cellular identities. Such reconstruction inevitably introduces model assumptions and algorithmic uncertainty, while lateral RNA diffusion during tissue lysis further degrades boundary fidelity between adjacent capture units, thereby reducing the effective spatial resolution (Figure 3b).

Microfluidic chip‐based spatial barcoding strategies can achieve pixel‐level resolutions on the order of 10‐50 µm (Figure 3a), offering improved spatial precision relative to conventional array‐based methods. Nonetheless, these approaches remain susceptible to spatial blurring arising from in situ molecular diffusion (Figure 3b). Similarly, single‐cell dissociation‐based spatial barcoding achieves single‐cell sequencing resolution but infers spatial positions indirectly, with effective spatial resolution constrained by barcode design (Figure 3). Photo‐directed spatial labelling and proximity‐linked molecular inference‐based strategies are likewise limited by the minimal achievable optical marking range and molecular interaction scale, respectively (Figure 3a).

In contrast, imaging‐based spatial omics methods directly localize individual molecules in situ, enabling precise delineation of cellular and subcellular structures without reliance on spatial averaging or post hoc deconvolution. This direct spatial readout allows high‐fidelity mapping of cell‐cell interactions, rare cell populations, and localized microenvironmental niches, preserving native tissue architecture and molecular context (Figure 3a). Nevertheless, these approaches may be affected by cumulative signal loss and alignment errors introduced by multi‐round imaging, including photobleaching, imperfect registration, and decoding inefficiency in densely labelled regions (Figure 3b). As a result, imaging‐based approaches provide a fundamentally different mode of spatial resolution, rooted in physical localization rather than computational inference.

From a methodological standpoint, NGS‐based spatial omics platforms benefit from highly automated workflows that support large‐area tissue profiling and scalable data generation. However, their performance is intrinsically constrained by algorithmic bias, dependence on high‐quality reference datasets, and the substantial costs associated with high‐density array fabrication, deep sequencing, and 3D multi‐slice reconstruction. Among these limitations, RNA diffusion during tissue permeabilization and lysis represents a fundamental source of spatial bias, leading to transcript mislocalization, compromised cell‐type inference, and attenuation of true biological heterogeneity. This discrepancy is far greater than what was indicated during the methodological validation [120].

By contrast, imaging‐based spatial omics approaches preserve intact tissue volumes and native spatial relationships, enabling accurate reconstruction of tissue microenvironments and cellular organization. This advantage is particularly critical for studying complex tissue architectures, irregular cell morphologies, localized signalling processes, and dynamic gene expression patterns. At single‐cell and 3D scales, imaging‐based platforms achieve high‐confidence spatial resolution through the integration of experimental structural markers with advanced computational analysis, whereas NGS‐based approaches typically rely on serial tissue sectioning and indirect reconstruction to infer 3D spatial organization.

### 3.3 Expansion of the Information Dimensions: Multimodality Detection

**FIGURE 4:**
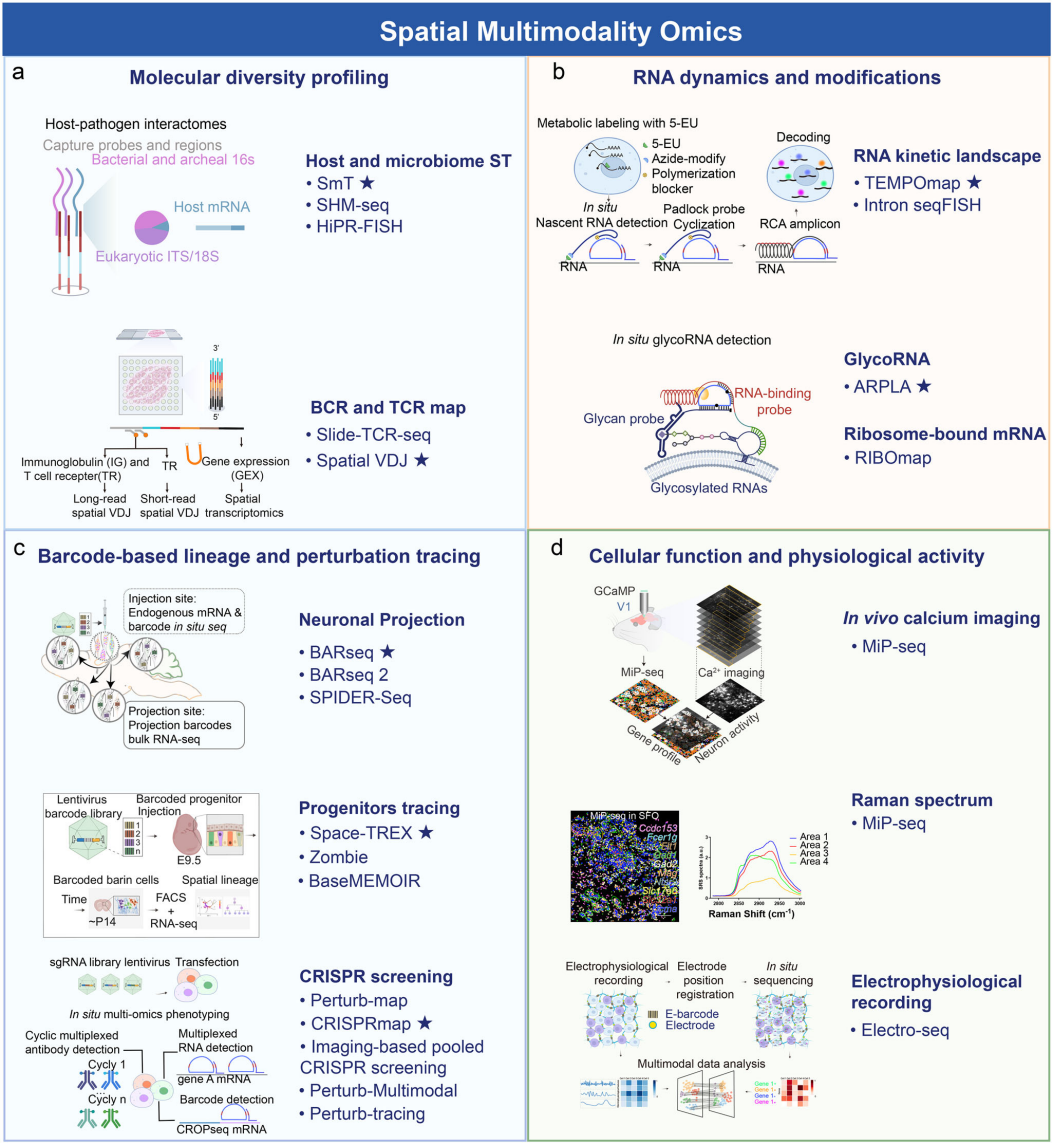
Multimodal applications of spatial omics techniques. Spatial multi‐modal omics integrates molecular, lineage, and functional information to link spatial gene expression with cellular dynamics and behavior. (a) Molecular diversity profiling enables spatial co‐detection of host and pathogen transcriptomes using SmT, SHM‐seq, and HiPR‐FISH, while immune repertoire diversity is resolved by Slide‐TCR‐seq and Spatial VDJ sequencing. (b) RNA dynamics and molecular modifications are captured by approaches such as TEMPOmap and intron seqFISH for nascent RNA tracking, ARPLA for in situ visualization of glycosylated RNA, and RIBOmap for spatial ribosome profiling. (c) Barcode‐based lineage and perturbation tracing reconstructs cellular ancestry and causal gene function in situ, including neuronal projection mapping by BARseq, BARseq2, and SPIDER‐Seq, lineage reconstruction by Space‐TREX, Zombie, and baseMEMOIR, and spatially resolved CRISPR perturbation phenotyping by Perturb‐map, CRISPRmap, imaging‐based pooled CRISPR screening, Perturb‐Multimodal, and Perturb‐tracing. (d) Functional and physiological integration links spatial molecular states to cellular activity, exemplified by MiP‐seq combining spatial transcriptomics with in vivo calcium or Raman imaging, and electro‐seq integrating spatial expression with chronic electrophysiological recordings. For multimodal spatial omics subtypes whose schematics cannot be uniformly displayed, representative methods are indicated by asterisks. Schematic diagrams for SmT [33], TEMPOmap [69], Space‐TREX [59], and MiP‐seq [75] were adapted with permission (Copyright Springer Nature). Schematics for Spatial VDJ [34], ARPLA [72], BARseq [123], CRISPRmap [76], and electro‐seq [68] were created by the authors based on the referenced conceptual frameworks.

Recent advances in multimodal spatial omics have substantially expanded the informational dimensions accessible within intact tissues, enabling the integration of molecular, functional, and phenotypic readouts within a unified spatial framework (Figure 4). These developments can be broadly organized into four complementary dimensions: molecular diversity profiling, RNA dynamics and modifications, barcode‐based lineage and perturbation tracing, and cellular function and physiological activity. First, expansion along the molecular diversity dimension has enabled spatially resolved profiling beyond canonical poly(A) mRNA expression. Optimized dual‐transcriptome capture strategies allow high‐resolution mapping of host–microbe interfaces, including microbial phylogenetic diversity and spatial immune repertoire dynamics via TCR/BCR sequencing [33, 34, 121] (Figure 4a). Second, spatial omics has increasingly captured molecular dynamics rather than steady‐state abundance. Advances in spatial ribosome profiling [70], RNA metabolic labelling [69], and glycosylation modification analysis [72], provide direct insight into spatiotemporal regulatory processes (Figure 4b). Thirdly, the integration of barcoding‐based lineage tracing and genetic perturbation has enabled causal and developmental inference in spatial contexts. Spatially resolved CRISPR screens and viral barcode tracing reconstruct gene regulatory functions, cellular genealogies, and neuronal projections within intact tissues (Figure 4c) [32, 59, 76, 116, 117, 122] (Figure 4c). Finally, spatial omics is increasingly integrated with functional and behavioural modalities, linking molecular states to physiological outputs. The coupling of spatial transcriptomics with functional imaging, electrophysiology, calcium dynamics, and vibrational imaging modalities enables direct correlation between molecular spatial patterns and cellular activity [58, 68, 75] (Figure 4d). Emerging integrations with optogenetics and intracellular state sensors (e.g., pH, redox, and voltage indicators) will further extend spatial omics toward real‐time functional interrogation.

Collectively, these multimodal integrations transform spatial omics from descriptive molecular mapping into a comprehensive framework for linking molecular architecture, dynamic regulation, lineage history, and functional phenotypes across biological systems.

### 3.4 Performance Trade‐Offs and Opportunities for Improvement

#### 3.4.1 NGS‐Based Spatial Omics: Throughput and Scalability vs. Resolution Fidelity

NGS‐based spatial omics platforms are designed for automated, high‐throughput profiling across large tissue areas, making them particularly well suited for discovery‐driven studies. These technologies provide broad molecular coverage, enabling transcriptome‐wide expression analysis and extensions to chromatin accessibility, epigenetic regulation, and immune repertoire profiling. Recent advances have further expanded their multimodal scope, allowing integration with lineage tracing, functional imaging, and CRISPR‐based spatial perturbation screens.

However, these strengths are intrinsically coupled to two major constraints: limited capture efficiency and restricted effective spatial resolution. Reduced sensitivity arises from poly(A)‐dependent capture, amplification bias, inefficient reverse transcription, and finite sequencing depth, disproportionately affecting low‐abundance transcripts and transient molecular states. Effective spatial resolution is further compromised by RNA diffusion during tissue permeabilization and lysis, dissociation‐related artifacts, and signal averaging within individual capture units [124]. Consequently, most NGS‐based platforms cannot directly resolve single‐cell or subcellular organization and instead rely on computational deconvolution or external imaging references.

These limitations are tightly linked. Reduced capture efficiency amplifies uncertainty in deconvolution‐based cell‐type inference, while diffusion‐driven transcript mislocalization introduces systematic spatial bias that obscures intrinsic genuine biological heterogeneity. As a result, NGS‐based spatial omics face an inherent trade‐off between molecular breadth, sensitivity, and spatial resolution fidelity. Ongoing improvements aim to enhance capture efficiency, refine spatial inference, and expand detectable molecular modalities, but this trade‐off remains a defining feature of the approach.

#### 3.4.2 Imaging‐Based Spatial Omics: Resolution Fidelity and Functional Integration vs. Target Coverage

Imaging‐based spatial omics technologies are intrinsically optimized for high detection efficiency and true subcellular spatial resolution [125]. By directly localizing individual molecules in situ, these approaches preserve native tissue architecture and enable precise delineation of cellular boundaries, subcellular compartments, and local microenvironments. This capability supports high‐fidelity mapping of cell‐cell interactions, rare cell populations, and spatially restricted signaling processes without reliance on spatial averaging or computational reconstruction. A defining strength of imaging‐based platforms is their compatibility with functional and perturbative modalities. Integration with genetic manipulation, calcium imaging, or electrophysiological measurements enables direct connection between molecular states and cellular activity, providing mechanistic insight that is difficult to achieve with NGS‐based methods alone.

Imaging‐based approaches, however, face complementary limitations. Probe‐dependent detection restricts analysis to predefined targets, limiting unbiased discovery. In addition, optical crowding, signal overlap, and cumulative decoding errors constrain accuracy and scalability, particularly in dense or 3D tissues. Imaging throughput and tissue penetration therefore remain practical bottlenecks, despite rapid advances in probe design, decoding algorithms, tissue clearing, and automated volumetric imaging.

#### 3.4.3 Complementarity Rather Than Competition

NGS‐based and imaging‐based spatial omics technologies are best viewed as complementary rather than interchangeable. NGS‐based platforms excel in large‐scale, automated tissue profiling with broad molecular coverage, supporting exploratory and hypothesis‐generating studies. Imaging‐based approaches provide superior spatial resolution and detection fidelity, enabling targeted analyses that require precise cellular or subcellular localization and direct integration with functional readouts. In practical settings, these complementarities are particularly pronounced. Discriminating highly similar, low‐abundance cell states, such as malignant neural progenitor‐like cells vs. non‐malignant neurons, cannot be reliably achieved using spot‐level NGS‐based platforms alone (for example, 10x Visium). Instead, integration with imaging‐based spatial proteomics, such as CODEX with multiplexed antibody panels, is required to achieve subcellular or single‐molecule resolution. Meanwhile, the multimodal framework enables high‐fidelity reconstruction of transient molecular states within their native spatial context, thereby mitigating the intrinsic resolution and sensitivity constraints of spot‐based transcriptomic approaches [126]. In addition, integrating single‐cell sequencing with imaging‐based targeted spatial architectures represents an alternative optimal strategy. Accordingly, the selection of spatial omics technologies should be guided by biological questions rather than by platform‐centric considerations. Strategic integration of NGS‐based and imaging‐based approaches will be essential for achieving comprehensive, multiscale understanding of tissue organization, cellular interactions, and dynamic biological processes in space.

## 4 Computational Bottlenecks and Emerging Solutions in Spatial Omics

The rapid expansion of spatial omics technologies has been paralleled by an explosion of computational methods, yet data analysis remains a central bottleneck due to the intrinsic complexity, sparsity, and scale of spatially resolved molecular measurements [127]. A primary challenge lies in data heterogeneity: spatial omics datasets differ substantially in modality, spatial resolution, coordinate systems, and noise structure, rendering cross‐platform integration nontrivial [128, 129]. Existing strategies, including landmark‐based registration, reference‐guided mapping, and shared feature alignment, improve comparability but remain highly sensitive to batch effects, incomplete marker overlap, and tissue deformation, thereby propagating uncertainty into downstream analyses. Compounding this issue, low capture efficiency, molecular diffusion, and optical crowding introduce spatially structured sparsity and dropout, violating the independence assumptions underlying many imputation models. While cross‐modal and foundation‐model‐based approaches can partially recover missing signals, they risk oversmoothing and obscuring fine‐grained spatial microdomains, making it difficult to distinguish true biological absence from technical loss [130].

Accurate cell segmentation represents another critical and unresolved challenge: errors in delineating cellular boundaries, particularly in dense tissues, irregular morphologies, or 3D volumes, propagate nonlinearly into cell‐type annotation and spatial interaction inference [131]. Although deep learning‐based segmentation methods have improved performance, their dependence on tissue type, imaging modality, and training data limits robustness and generalizability. At a higher level of inference, extracting biologically meaningful cell‐cell interactions from spatial proximity remains conceptually challenging. Many current spatial statistics rely on distance‐based or neighbourhood enrichment metrics that lack mechanistic grounding and are sensitive to sampling density and tissue architecture, underscoring the need for models that integrate molecular states, temporal dynamics, and perturbation information.

These analytical challenges are further exacerbated by scalability constraints: high‐resolution imaging and large NGS‐based spatial datasets impose substantial demands on storage, memory, and computation, and while cloud‐native infrastructures, compressed data formats, and GPU acceleration offer partial relief, scalable algorithms that preserve spatial fidelity remain limited. Finally, the increasing reliance on deep learning raises concerns regarding interpretability and validation, as black‐box predictions without uncertainty quantification or experimental perturbation risk overinterpretation. Addressing these challenges will require probabilistic and mechanistically informed models that explicitly incorporate physical constraints, uncertainty, and causal perturbations, alongside interoperable data standards and modular software ecosystems to enhance reproducibility, cross‐study integration, and biological interpretability.

## 5 Applications in Biomedicine

Spatial omics technologies are revolutionizing biomedical research by enabling comprehensive, multidimensional analysis of cellular systems within their native tissue context. They provide high‐resolution spatial mapping of biomolecules to reveal cellular heterogeneity and microenvironmental organization, integrated multi‐omics profiling (genomics, transcriptomics, proteomics) to decipher complex regulatory architectures, and temporal dynamics analysis via serial sampling and pseudotime reconstruction. Such fundamental capabilities have proven particularly transformative in biomedical research on tumorigenesis (characterizing microenvironmental niches), embryonic developmental process (tracing lineage specification), brain architecture (mapping neural circuits), and infection mechanism (resolving host‐pathogen interactions) [3, 132, 133, 134, 135]. By systematically correlating molecular signatures with their spatial and temporal contexts and functional dynamics, spatial omics has emerged as an indispensable platform for both mechanistic biomedical discovery and translational applications.

By enabling multidimensional characterization of cellular interactions and molecular profiles, spatial omics technologies have significantly advanced our understanding of tumor microenvironment (TME) dynamics. Building upon single‐cell sequencing discoveries of TME heterogeneity [136, 137], spatial omics analysis reveals how tumorigenesis drives spatial reorganization of diverse cell populations. Furthermore, these analysis have yielded several key insights into tumorigenesis, including compartmentalization of exhausted immune cells within tumor parenchyma vs. dynamic tumor‐immune interactions at invasive margins [52], and distinct spatial correlations between stromal cells and tertiary lymphoid structures [138]. With ongoing improvements in spatial resolution, detection sensitivity, and clinical translation [139], these technologies are poised to transform precision oncology by elucidating the spatiotemporal evolution of tumor ecosystems across diverse molecular dimensions (transcriptomic, proteomic, epigenomic, and metabolomic) [140].

Spatial omics technologies have become indispensable for elucidating the spatiotemporal regulation of embryonic development, bridging the critical gap between transcriptional heterogeneity and physical and anatomical organization. These approaches including Geo‐seq [14], DBiT‐seq [35], Stereo‐seq [26], and MISAR‐seq [36] enable multidimensional mapping of developmental processes by simultaneously capturing transcriptomic and epigenomic information within native spatial contexts. Geo‐seq pioneered this field by generating the first spatially resolved transcriptomic atlas of mouse embryogenesis [14], while DBiT‐seq and MISAR‐seq revealed how spatial epigenetic landscapes direct cellular differentiation [35, 36]. The mouse organogenesis spatiotemporal atlas (MOSTA), constructed using Stereo‐seq technology, systematically documented transcriptional changes during organ formation [26]. And sci‐Space identified thousands of spatially patterned genes that define cellular migration trajectories [57]. These technologies have deepened our understanding of molecular architectures underlying spatial cellular heterogeneity, microenvironmental regulation of cell fate decisions, and dynamic behaviour of gene regulatory networks. As these methods rapidly evolve, their application to developmental perturbations will uncover the etiology of congenital disorders, thereby creating new synergies between developmental biology and clinical medicine.

Spatial omics technologies are integrating macroscopic neuroanatomy with microscopic molecular profiles by enabling multiscale analysis of the brain's intricate cellular architecture and connectivity, thereby achieving unprecedented resolution in delineating neural circuits and cellular states. The pioneering work from Anthony Zador's lab integrated neural circuit delineation with microscopic molecular profiles at single‐neuron resolution via BARseq, a method combining MAPseq (high‐throughput mapping of projection networks using viral RNA barcodes [141]) with in situ sequencing [123]. The advanced BARseq2 platform further integrated projection tracing with multiplexed gene expression profiling [142], revealing circuit‐specific molecular signatures. Beyond neural connectivity mapping, spatial omics technologies aid in identifying neurodegenerative disease‐associated cell types and biomarkers through spatially resolved molecular profiling. In addition, spatial omics technologies preserve neuronal morphology while enabling thick‐tissue analysis, facilitating investigations into synaptic plasticity, circuit interactions, and degenerative mechanisms [143]. By correlating molecular profiles with anatomical and functional connectivity, spatial omics technologies provide a powerful framework for understanding the brain's organizational principles across diverse species.

Spatial omics technologies can resolve the spatiotemporal dynamics of infection processes, from microbial proliferation to immune evasion strategies, contributing to the understanding of host‐pathogen interactions. These approaches have mapped immune gene expression patterns across development stages of Mycobacterium tuberculosis granuloma, revealing cell‐type‐specific responses through analysis of 34 immune‐related genes in murine models [144]. Recent methodological advances enable comprehensive host‐microbe co‐profiling, as demonstrated by enhanced Visium's application in COVID‐19 research, where dual transcriptome analysis of SARS‐CoV‐2 and host cells in FFPE lung tissues identified critical spatial virus‐host cell interaction sites [145].

## 6 Overcoming Current Limitations: Future Directions in Spatial Omics

Since the emergence of spatial omics technologies, hundreds of methods have been developed, with nearly one new spatial omics analysis tool being released on GitHub every week. This unprecedented momentum has driven rapid advancements in the field. However, several persistent challenges hinder their widespread adoption in biomedical research, including challenges from technical operation, data analysis, and clinical translation. Accordingly, we will propose corresponding strategies to address the various challenges below.

Among technical operation challenges, the persistent trade‐off among spatial resolution, multiplexing capacity, and cost remains a fundamental constraint. Addressing this trade‐off primarily depends on advances in experimental design and data quality, including improved probe chemistry, optical performance, and sample preparation. Moreover, deep learning‐based approaches can serve as complementary tools to enhance image quality and data interpretation when applied to robust experimental datasets. In this broader context, mass spectrometry‐based spatial proteomics and metabolomics have emerged as an important and complementary modality, offering label‐free, highly multiplexed molecular detection that is not constrained by predefined probes. Although these approaches currently face limitations in spatial resolution, throughput, and accessibility, ongoing advances in instrumentation, sample preparation workflows, and ionization strategies are steadily improving their performance and scalability.

Lack of standardization across platforms impedes reproducibility, spurring efforts to establish standard operating procedures (SOPs) and quality control frameworks. Furthermore, multi‐omics integration on single tissues, including the combination of transcriptomic, proteomic, and metabolomic layers, demands co‐optimized sample preparation and computational integration strategies. Finally, sensitivity limitations, particularly in detecting low‐abundance molecules, are being progressively addressed through enhanced probe design, enzymatic amplification, sequencing saturation, and, in parallel, improvements in mass spectrometry sensitivity and data acquisition efficiency.

During data analysis, integrating data across platforms and modalities relies on molecular anchoring and landmark‐aware registration algorithms (e.g., SOAPy [146]). Missing data and sparsity issues are increasingly addressed using cross‐modal imputation models (e.g., scGPT‐spatial [147]). Standardization efforts are advancing through open‐source ecosystems such as SpatialData [127] and OME‐Zarr [148], which provide unified data architectures. Computational bottlenecks posed by data scale are being alleviated via compressive sensing techniques and cloud‐native solutions, significantly improving processing efficiency.

Clinical translation is challenged by high costs for large‐scale clinical adoption. Target and region pre‐screening based on histopathological and multi‐omics data can help improve cost‐effectiveness. Meanwhile, clinical translation is facing reproducibility, robustness, and validation challenges, which require clinically compliant SOPs and multi‐center prospective studies to demonstrate utility. Moreover, extracting biologically and clinically meaningful insights necessitates collaborative interpretation across computational, biological, and clinical domains, supported by AI‐assisted annotation tools.

Spatial omics technologies are undergoing rapid transformation through technological innovation and computational integration. The concurrent advancement of high‐resolution light‐sheet microscopy, expansion microscopy, tissue clearing, synthetic molecular probes, high‐throughput fluorescence lifetime imaging, and AI algorithms has collectively facilitated the emergence of whole‐tissue 3D spatial multi‐omics platforms. These platforms overcome the limitations of traditional 2D methods by integrating high‐resolution and high‐throughput profiling with continuous sampling, thereby facilitating comprehensive mapping of molecular architecture across both physiological and pathological contexts.

When integrated with machine learning analysis of perturbation atlases, these advances promise to establish new paradigms for developmental biology, disease mechanisms, and therapeutic discovery. The convergence of artificial intelligence with perturbation studies, including genetic, environmental, and pharmacological interventions, is driving life sciences toward more precise and efficient methodologies. Virtual cell modelling tools (e.g., STATE [149], CAX [150], cell behaviour hypothesis grammar [151], AIVC [152], and Arc Virtual Cell Atlas [153]) and large‐scale initiatives like the CAS Human Cell Lineage Facility are revolutionizing traditional research approaches. As spatial omics datasets, such as those in glycomics and exosome research, continue to expand, they accelerate the decoding of the “multilingual system” of intercellular communication. This growth enhances the dimensionality, fidelity, and predictive power of computational models in the field. Such computational platforms will not only enable large‐scale simulations to reduce experimental burden but also provide novel insights for drug screening, tumor microenvironment analysis, and developmental biology studies, positioning them as foundational methodologies for next‐generation biomedical research.

## 7 Conclusion

Recent advances in spatial omics technologies have substantially enhanced spatial resolution, detection throughput, and multi‐omics integration through continuous innovation in both NGS and imaging‐based platforms. These technological innovations have enabled diverse applications, including spatial expression profiling, chromatin accessibility mapping, live‐cell dynamics, and lineage tracing, thereby deepening our understanding of tumorigenesis, development process, brain connectivity/function, and pathogen infection. Furthermore, integration with functional and phenotyping techniques, such as calcium imaging and Raman spectroscopy, continues to expand the analytical capacities of spatial omics.

NGS platforms have significantly improved their technical versatility by increasing spot resolution, raising cluster density, optimizing in situ capture modalities, reducing chip fabrication costs, expanding spatial coverage, and broadening capture‐based multi‐omics and multi‐modal applications. Future development efforts should focus on further enhancing spatial resolution and capture efficiency. In parallel, imaging‐based platforms have achieved performance gains through optimized probe design, advanced decoding strategies, improved signal amplification methods, higher operational efficiency, and extended support for multi‐omics and multi‐modal analyses. Further increasing detection throughput remains a priority for imaging‐based platforms. Spatial omics technologies are evolving to meet diverse, multi‐level needs. Investigations of fine‐grained biological processes like development and disease require integrative, multi‐omic analyses at single‐cell resolution. In contrast, clinical translation demands robust, cost‐effective, and scalable solutions, typically employing targeted panels. Defining standardized operating procedures for these different tiers of application is a pressing challenge.

Despite these advancements, the field continues to face persistent challenges, which can be categorized into technical operations, data analysis, and clinical translation. Addressing these challenges requires a multifaceted strategy. Therefore, we propose an integrated framework designed to: optimize the trade‐off between spatial resolution, throughput, and cost through deep learning and targeted regional analysis; improve spatial data analysis by constructing unified data architectures and scalable cloud‐computing solutions; and accelerate clinical translation via strategies for cost‐effective pre‐screening and multi‐center validation. Together, these strategies help bridge technical, analytical, and interoperability gaps, laying a robust foundation for next‐generation spatial omics applications.

## Author Contributions

G.C., J.D. and X.W. contributed equally to funding acquisition. X.W. led the writing of the original draft. G.C. and J.D. provided major contributions to manuscript review and editing. W.X., L.D., and L.S. contributed to the preparation of the original draft. All authors reviewed and approved the final manuscript.

## Funding

This work was supported by the National Natural Science Foundation of China (Grant Nos. 32401246, 32171022, and 32221005) and The Special Funds Project for Strategic Emerging Industries of Shenzhen Municipal Development and Reform Commission (XMHT20240215002).

## Conflicts of Interest

The authors declare no conflicts of interest.

## Supporting information




**Supporting File**: advs74562‐sup‐0001‐DataFile.xlsx.

## Data Availability

The authors have nothing to report.
